# A Combined Solution and Solid-State Study on the Tautomerism of an Azocalix[4]arene Chromoionophore

**DOI:** 10.3390/molecules28124704

**Published:** 2023-06-12

**Authors:** Laura Baldini, Davide Balestri, Luciano Marchiò, Alessandro Casnati

**Affiliations:** Department of Chemistry, Life Sciences and Environmental Sustainability, University of Parma, Parco Area delle Scienze 17/a, 43124 Parma, Italy; davide.balestri@unipr.it (D.B.); luciano.marchio@unipr.it (L.M.); alessandro.casnati@unipr.it (A.C.)

**Keywords:** chromoionophores, calix[4]arenes, azo-phenol–quinone-hydrazone tautomerism, Ca^2+^ complexation

## Abstract

Azocalixarenes functionalized with cation binding sites are popular chromoionophores due to the ease of synthesis and the large complexation-induced shifts of their absorption band that originate from an azo-phenol–quinone-hydrazone tautomerism. Despite their extensive use, however, a thorough investigation of the structure of their metal complexes has not been reported. We describe herein the synthesis of a new azocalixarene ligand (**2**) and the study of its complexation properties with the Ca^2+^ cation. Through a combination of solution (^1^H NMR and UV-vis spectroscopies) and solid-state (X-ray diffractometry) techniques, we demonstrate that metal complexation induces a shift of the tautomeric equilibration towards the quinone-hydrazone form, while deprotonation of the complex results in the reversion to the azo-phenol tautomer.

## 1. Introduction

Chromoionophores are synthetic molecular receptors that combine in their structure an ionophoric site for the complexation of an ion analyte and a chromogenic moiety that changes color in response to the recognition event. Crucial for the correct functioning of chromoionophore-based sensors is the connection between the sensing and reporting units. In the case of chromoionophores for the optical detection of metal cations, a fast and strong response can generally be achieved if a portion of the chromogenic moiety takes part in the coordination site. In this way, upon complexation, the excited state of the chromophore can be stabilized or destabilized more strongly than the ground state, resulting in a bathochromic or hypsochromic shift, respectively, of the electronic absorption band [1].

Calixarene-based chromoionophores represent one of the first and most successful applications of calixarene chemistry. Thanks to the ease of functionalization of the two rims, the calixarene scaffold can be equipped with both cation binding sites and chromophoric reporter units that undergo a color change upon metal ion complexation [2,3,4,5,6,7,8,9].

Azocalixarenes, which have the phenylazo (-N=N-Ph) group linked to the para-position of one (or more) phenol rings, are among the most studied chromogenic calixarenes. The popularity of these compounds can be ascribed to the synthetically appealing one-step diazo-coupling reaction that, pioneered in 1989 by Shinkai [10], gives good results on the calixarene phenol rings. Moreover, in azocalixarenes, the resulting 4-phenylazophenol chromophore can directly participate in cation complexation with the phenol oxygen atom as a donor site. A vast number of azocalixarene chromoionophores have been reported, with different additional binding sites for cations and different numbers and functionalizations of the 4-phenylazophenol moieties [5,7,9,11,12,13,14,15,16,17,18,19,20,21].

**Figure 1:**
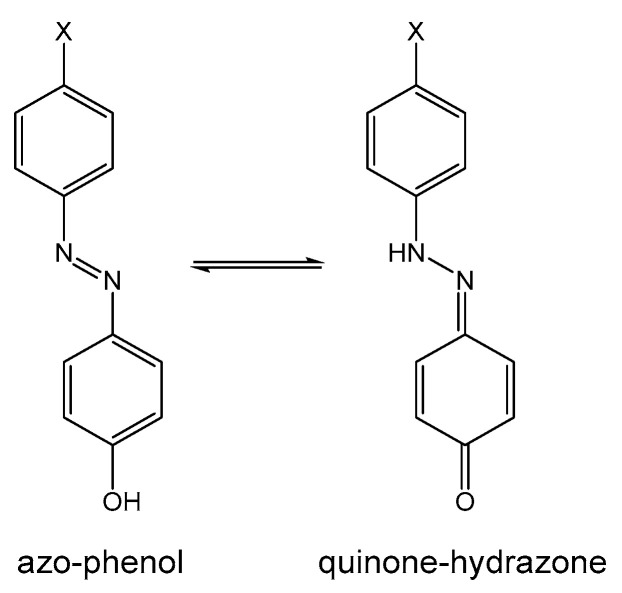
Azo-phenol–quinone-hydrazone tautomerism of 4-phenylazophenol.

The 4-phenylazophenol moiety is well known to undergo a tautomeric equilibration between the azo-phenol and quinone-hydrazone forms (Figure 1), which depends on the substituents of the phenyl ring and on the solvent [22]. Electron-donating substituents generally stabilize the azo-phenol tautomer, while electron-withdrawing ones favor the quinone-hydrazone form. Moreover, due to the stronger hydrogen-bonding ability of the OH group of the azo-phenol compared to the NH group of the quinone-hydrazone, hydrogen-bonding acceptor solvents (such as pyridine and acetone) usually stabilize the former, while hydrogen-bonding donor solvents (such as acetic acid and chloroform) favor the latter form.

The two tautomeric forms can be easily distinguished by UV-vis spectroscopy. The absorption band of the azo-phenol tautomer is typically displayed around 400 nm, while it is bathochromically shifted to ~480 nm for the quinone-hydrazone form [22,23,24,25].

When azocalixarenes form complexes with metal cations, large shifts of the absorption band of the azo-phenol moiety are observed, which have been attributed, in some cases, to a cation-induced stabilization of the quinone-hydrazone form [14,17,26,27] and, in others, to a metal-induced deprotonation of the ligand [18,21]. Despite their large use as chromoionophores, the characterization of the complexes is mostly achieved exclusively by UV-vis spectroscopy. In just a few papers, the complexes have also been studied by ^1^H NMR [7,13,21], and only one crystal structure of an azocalixarene metal complex is present in the literature [20].

To fill this gap, we report herein the synthesis of a new azocalixarene chromoionophore (**2**) and the thorough characterization of its complex with the Ca^2+^ cation both in solution and in the solid state. In **2**, the binding site for the cation at the calixarene lower rim is constituted by two phenoxyacetamide groups linked to two distal phenol rings, which provide two phenolic and two carbonyl oxygen donors, and by the two phenol OH groups of the 4-nitrophenylazophenol moieties. This coordination environment has been shown in the literature to be very efficient for the complexation of hard metal cations such as Mg^2+^ [8], Ca^2+^ [6,18], Fe^3+^ [28], or trivalent lanthanide ions [28,29]. Thanks to the combined use of UV-vis and ^1^H NMR spectroscopies and X-ray diffractometry, we have been able not only to confirm the high affinity of this ligand for the Ca^2+^ cation but also to shed light on the structure of the complex and on the tautomeric equilibration.

## 2. Results and Discussion

### 2.1. Synthesis and Characterization of ***2***

**Scheme 1:**
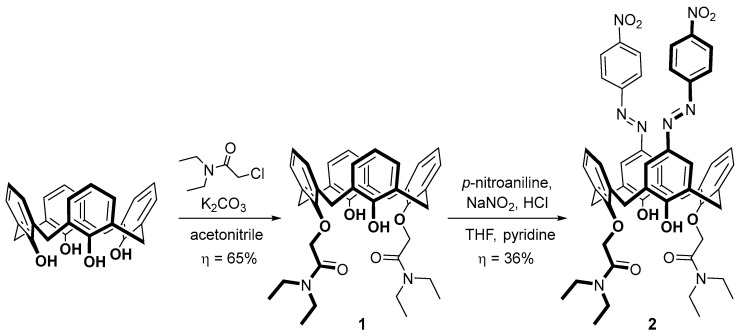
Synthesis of compound **2**.

Azocalixarene **2** was synthesized in only two steps, following the reaction pathway reported in Scheme 1. First, diamidocalix[4]arene **1** was obtained in good yield from the alkylation of calix[4]arene with 2-chloro-*N*,*N*-diethylacetamide, carried out according to the literature [30]. Subsequently, calixarene **1** was subjected to a diazonium coupling reaction with p-nitroaniline following a procedure adapted from [16].

**Figure 2:**
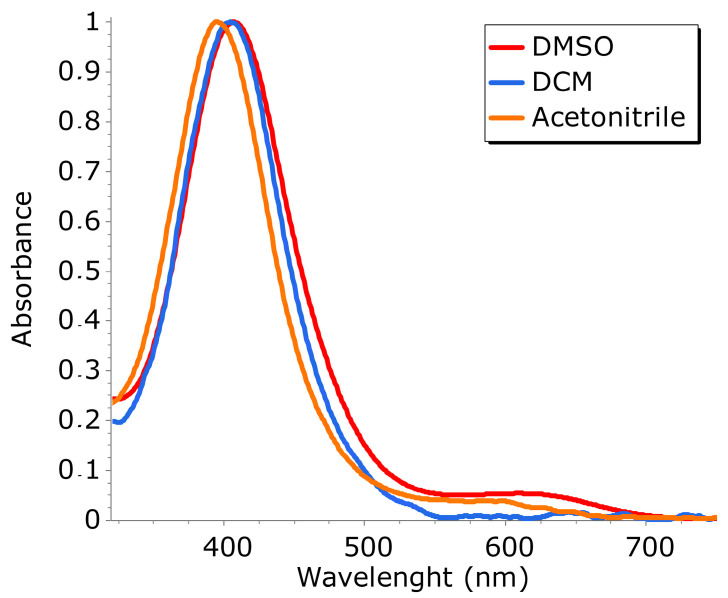
Normalized absorption spectra of **2** (1 × 10^−5^ M) in DMSO (red line), dichloromethane (blue line), and acetonitrile (orange line).

Figure 2 displays the absorption spectra of azocalixarene **2** in different solvents. Despite the presence of the electron-withdrawing nitro groups, both 4-nitrophenylazophenol moieties of **2** are present exclusively in the azo-phenol form, as indicated by the absorption band centered around 400 nm. The strong intramolecular hydrogen bonds that the phenol OH groups can donate either to the neighboring ethereal oxygen atoms or to the carbonyl C=O groups are most likely responsible for the stabilization of the azo-phenol tautomer. Similar calix[4]arene derivatives containing two 4-nitrophenylazophenol moieties in a distal position display analogous absorption spectra, confirming the shift of the tautomeric equilibration towards the azo-phenol form when this unit is embedded in the calixarene skeleton [12,21].

**Figure 3:**
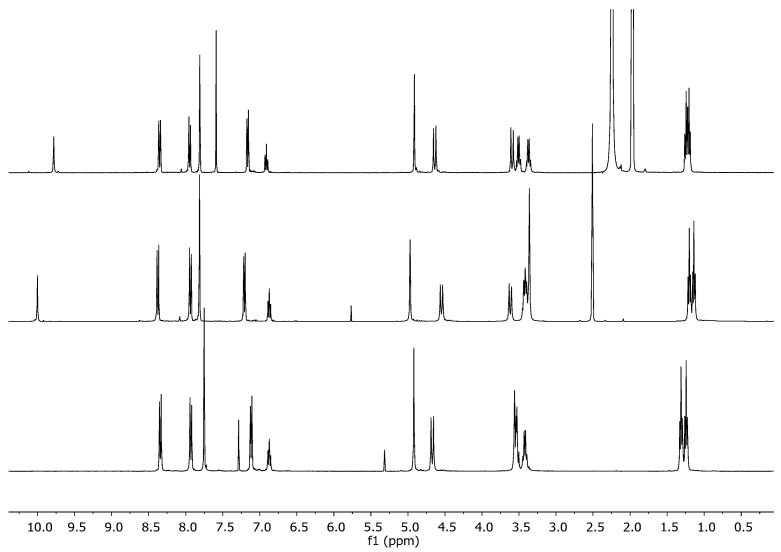
^1^H NMR spectra of **2** in different solvents. From top to bottom: CD_3_CN-CDCl_3_ 5:1 (*v/v*), DMSO-*d*_6_, CDCl_3_.

^1^H NMR spectroscopy (Figure 3) shows that in acetonitrile, DMSO, and chloroform, calixarene **2** adopts the cone geometry, with the typical AX pattern of the axial and equatorial protons of the methylene bridges around 4.5 and 3.5 ppm, respectively. The signals of the hydrogen-bonded OH protons around 9.8 ppm in acetonitrile and DMSO confirm the sole presence of the azo-phenol tautomer. In chloroform, where the OH signal is missing due to chemical exchange, the same form is suggested by the invariance of all the signals with respect to those in acetonitrile and DMSO solutions.

**Figure 4:**
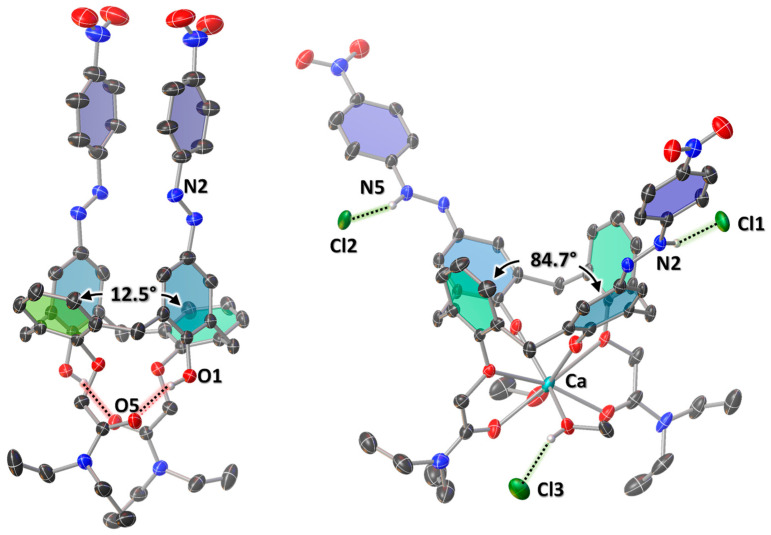
Molecular structure of **2** (**left**) and of **2∙CaCl_2_** (**right**). Thermal ellipsoids are depicted at the 30% probability level.

The X-ray structure analysis of crystals of **2** grown from a mixture of chloroform, methanol, and hexane (Figure 4, left) shows a flattened cone conformation of the calixarene, with the phenol rings of the phenylazophenol moieties almost parallel, having a dihedral angle of 12.5°, and with a minimum distance between the nitrophenyl rings of 3.68 Å, indicative of weak π–π interaction between the two aromatic moieties. The two phenol rings functionalized with the amide groups are tilted outward, having a dihedral angle of 67.4°. This conformation is stabilized by two strong hydrogen bonds between the phenol OH groups and the amide C=O groups (O1···O5 of approximately 2.62 Å). In the Ar-OH aromatic ring, the C-C distances vary in the 1.38–1.41 Å range, the C-O distance is 1.35 Å, and the N-N distance of the azo moiety is 1.24–1.29 Å (by taking into account the two disordered fragments, Figure S12). Taken together, these geometric parameters are suggestive of a single Ar-O bond and a double N=N bond for the azo group, pointing to the azo-phenol tautomer also in the solid state.

Interestingly, in the crystal packing, the nitro groups exchange O···HC interactions with the amide methyl groups of a neighboring molecule, thus giving rise to supramolecular chains that run parallel to the *b* crystallographic axis, as shown in Figure S6.

### 2.2. Ca^2+^ Complexation Studies

**Figure 5:**
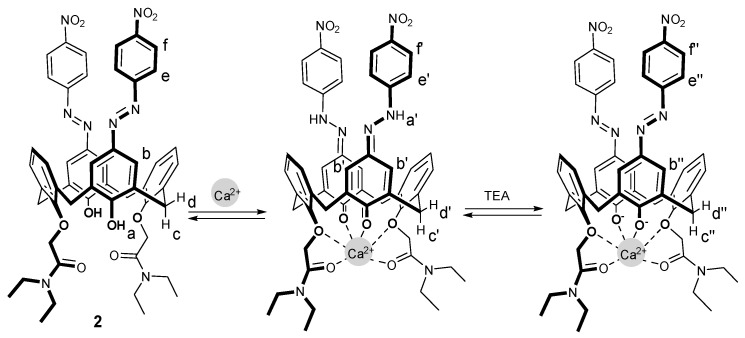

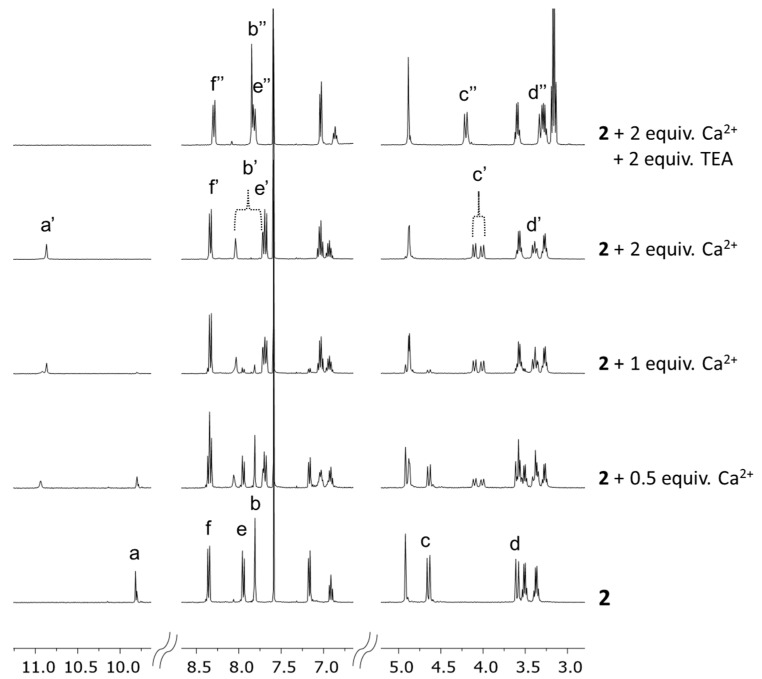
Partial ^1^H NMR spectra (400 MHz, CD_3_CN-CDCl_3_ 6:1, *v/v*) of ligand **2** (1.4 mM) upon addition of (from bottom to top) 0, 0.5, 1, 2 equiv. of Ca(ClO_4_)_2_, and 2 equiv. of Ca(ClO_4_)_2_ + 2 equiv. of TEA. For the assignment of the peaks to protons a-f, a′–f′ and b′′–f′′ cfr. the calixarene structures at the top of this figure.

The complexation properties of chromoionophore **2** for the Ca^2+^ cation were investigated in acetonitrile solution by ^1^H NMR titrations (Figure 5), with the aim of shedding light on the possibility of a metal ion-induced azo-phenol–quinone-hydrazone tautomerism or metal-induced deprotonation of the ligand. The addition of increasing amounts of Ca(ClO_4_)_2_ to a CD_3_CN-CDCl_3_ (6:1, *v/v*) solution of **2** resulted in the appearance of a new set of resonances consistent with the Ca^2+^ complex of ligand **2**, accompanied by the gradual disappearance of the signals of **2**. In the titration conditions, the complexation equilibrium is slow compared to the NMR timescale. After the addition of 1 equivalent of Ca^2+^, the signals of free **2** are barely visible, while they have completely disappeared in the presence of 2 equivalents of the guest. This quasi-saturation behavior is indicative of strong binding of the cation by receptor **2**, with a binding constant that can be estimated as >10^4^ M^−1^.

Besides assessing the efficiency of the complexation process, however, this experiment is particularly informative regarding the structural features of the complex and the tautomeric equilibrium. Three resonances of ligand **2** undergo significant modifications upon complex formation: (i) the signal of the phenol OH, originally seen at 9.82 ppm (a in Figure 5), is shifted to 10.87 ppm (a′); (ii) the singlet of the aromatic protons ortho to the azo group (b) is split into two signals (b′), one downfield shifted by 0.23 ppm and the other upfield shifted by 0.09 ppm; (iii) the doublet of the axial methylene bridge protons at 4.65 ppm (c) is split into two new doublets (c′), both significantly upfield shifted (Δδ = 0.54 and 0.64 ppm). Although similar shifts upon metal complex formation have been previously reported for analogous chromoionophores [7,13,21] and assigned to the non-symmetrical structure of the complex, with the cation occupying a lateral position of the binding cavity [7,13], in our opinion they are clearly indicative of the shift of the tautomeric equilibration towards the quinone-hydrazone moiety: (i) the hydrazone NH proton (a′), more deshielded than the phenol OH, gives rise to the singlet at 10.87; (ii) the C=N double bond is responsible for the loss of symmetry of the quinone rings and the consequent splitting of the resonances of the quinone protons (b′) that are now no longer equivalent; (iii) for the same reason, the four axial protons of the methylene bridges (c) become chemically non-equivalent in pairs and resonate as two separate doublets (c′) at a chemical shift around 4.0 ppm, which is typical for calix[4]arene diquinones [9,31].

The addition of 2 equivalents of triethylamine to the last sample of the NMR titration resulted in a color change from orange to deep purple and in modifications of the spectrum compatible with the deprotonation of the quinone-hydrazone moieties and with the consequent shift of the tautomeric equilibration back to the azo form: (i) the signal of the hydrazone NH proton (a′) disappears; (ii) the two peaks of the quinone ring at 8.04 and 7.71 ppm (b′) are replaced by a single resonance at 7.85 (b″); (iii) the signals of the methylene bridge revert to the typical AX doublets (c″ and d″). Overall, the spectrum of the deprotonated complex is characterized by the same pattern of signals as the spectrum of free **2**, with small shifts due to the presence of the complexed cation.

**Figure 6:**
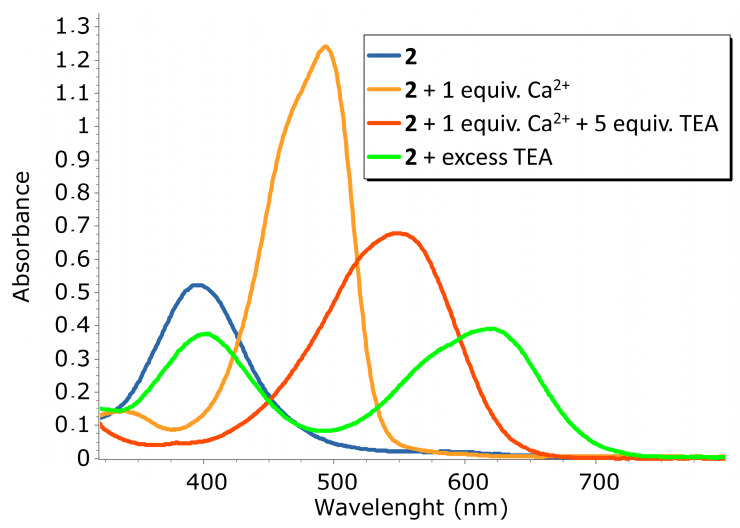
UV-vis spectra of ligand **2** (1 × 10^−5^ M in CH_3_CN, blue line) and ligand **2** (1 × 10^−5^ M in CH_3_CN) upon addition of 1 equiv. of Ca(ClO_4_)_2_ (orange line), 1 equiv. of Ca(ClO_4_)_2_ and 5 equiv. of TEA (red line), and ~50,000 equiv. of TEA (green line).

UV-vis spectroscopy (Figure 6) confirmed the metal complexation-induced tautomerism of the azophenol moieties: upon addition of one equivalent of Ca(ClO_4_)_2_ to a 1 × 10^−5^ M acetonitrile solution of **2**, the absorption band centered at 397 nm was replaced by a new band having a maximum at 494 nm, a typical value for the quinone-hydrazone tautomer [22]. Further additions of Ca^2+^ did not produce any modification of the spectrum, indicating a K_a_ > 10^6^ M^−1^. The addition of 5 equivalents of TEA to the 1:1 (**2**:Ca^2+^) solution produced a bathochromic shift of the absorption maximum to 549 nm. Interestingly, the spectrum of a 1 × 10^−5^ M solution of **2** containing a large excess (~50,000 equiv.) of TEA contained two separate absorption bands, one with a maximum at 400 nm, corresponding to the azophenol moiety, and the other centered at 620 nm, due to the deprotonated azophenol group. In the absence of the cation, a weak base such as TEA is therefore able to deprotonate only one phenol group. As expected, the presence of the cation complexed at the calixarene lower rim enhances the acidity of the ligand [15,21]. The hypsochromic shift of the absorption band of the deprotonated complex (centered at 549 nm) with respect to the deprotonated azophenol in metal-free **2** (centered at 620 nm) is likely due to the presence of the cation, which hinders the negative charge delocalization from the Ar-O^−^ to the nitro group.

Single crystals of the Ca^2+^ complex of **2** were obtained from the slow evaporation of a 1:1 solution of **2** and CaCl_2_ in a mixture of chloroform, methanol, and hexane. In the complex, the cation is coordinated by the six oxygen atoms at the lower rim of the calixarene (the four Ar-O and the two C=O) and by two methanol molecules of crystallization (Figure 4, right). The metal geometry is distorted square antiprismatic, with Ca-O bond lengths in the 2.35–2.65 Å range (Figure S11).

As a consequence of the cation complexation, the conformation of the calixarene scaffold undergoes considerable modification. The two aromatic rings functionalized with the azo groups, which were almost parallel in **2**, in **2∙CaCl_2_** are tilted outwards with a dihedral angle of 84.7°. This conformational change is required to bring the phenol oxygen atoms at a shorter distance to the cation. As a result, the other two rings, which were divergent in **2**, became almost parallel (dihedral angle of 27.1°). The overall macrocycle conformation can be described as a flattened cone that is inverted in terms of the orientation of the calixarene aromatic rings with respect to the one displayed by **2**.

The most important feature of this structure, however, is the evidence that, also in the solid state, the complexation of the cation induces the shift of the tautomeric equilibration towards the quinone-hydrazone moiety. According to the difference Fourier map, the two N atoms of the azo groups (N2 and N5) are protonated, with the hydrazone NH hydrogen atoms hydrogen bonded to the Cl^−^ anions (N2···Cl1 3.24 Å and N5···Cl2 3.28 Å, Table S3). Moreover, the bond distances within the quinone-hydrazone units are indicative of a C=O double bond (1.23 and 1.24 Å) conjugated to the two C=C double bonds of the quinone ring (in the 1.33–1.35 Å range) and to the C=N bond (1.31 and 1.32 Å) of the hydrazone group (Figure S12). The conjugation, however, does not extend to the nitrophenyl ring, which exhibits bond lengths similar to those of **2**.

Attempts to crystallize **2** in the presence of both CaCl_2_ and triethylamine were unsuccessful. We, therefore, prepared the neutral **2**^2−^∙Ca^2+^ complex by treating a dichloromethane solution of **2** with a saturated water solution of Ca(OH)_2_. The organic phase, whose color had changed immediately from orange to deep purple, was divided into two batches and let evaporate to grow crystals in the presence of hexane for the first batch and methanol for the second. Both solutions yielded crystals suitable for X-ray diffractometry. The two structures (**2∙Ca-A** and **2∙Ca-B**, respectively), albeit similarly consisting of the doubly deprotonated calixarene bound to the metal cation, present some differences.

**Figure 7:**
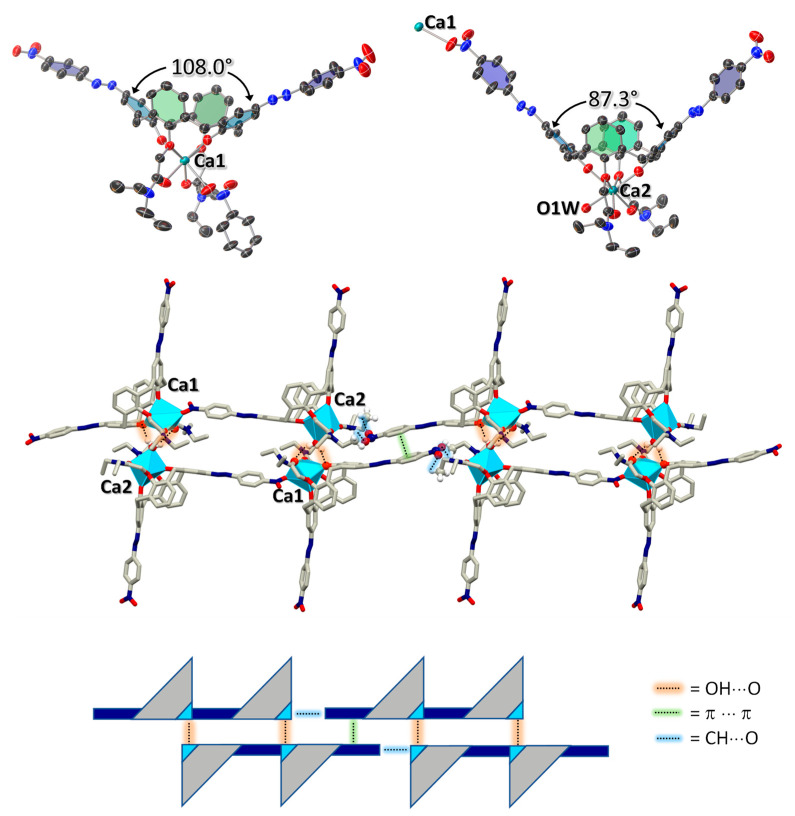
Top: molecular structure of the two complexes contained in the asymmetric unit for **2∙Ca-A** (thermal ellipsoids are depicted at the 30% probability level). Middle: packing view and weak interactions between **2∙Ca-A** dimers. Bottom: schematized view of weak supramolecular interactions.

The asymmetric unit of **2∙Ca-A** comprises two calixarene units and two calcium cations that exhibit a slightly different coordination environment (Figure 7). Both cations (Ca1 and Ca2) are coordinated by the six oxygen atoms at the lower rim of the calixarene (the four Ar-O and the two C=O), but while the seventh coordination site of Ca1 is occupied by an oxygen atom of a nitro group of the second calixarene moiety, Ca2 coordinates a water molecule instead (Figure S11). The metal geometry for both cations is distorted pentagonal bipyramidal, with Ca-O bond lengths in the 2.17–2.57 Å range. The shortest Ca-O distances are found between the metal and the deprotonated oxygen atoms of calixarene (2.17–2.25 Å range). The analysis of the bond lengths within the phenolate moiety (Figure S13) is consistent with the possible resonance forms that can accommodate the negative charge (Scheme S1). The N-N distances (in the range 1.21–1.29 Å) are consistent with a double N=N bond and support the NMR evidence that, upon deprotonation, the tautomeric equilibration reverts to the azo-phenol form.

The presence of the water molecule bound to Ca2 leads to the formation of a supramolecular tetramer sustained by two hydrogen bonds per water molecule (Figure S10). One of the nitrophenyl groups gives rise to a CH···O interaction with the aliphatic chains of an adjacent amide group and a π–π interaction with a symmetry-related aromatic ring. Overall, the system forms a supramolecular structure formed by two antiparallel chains (Figure 7, bottom).

**Figure 8:**
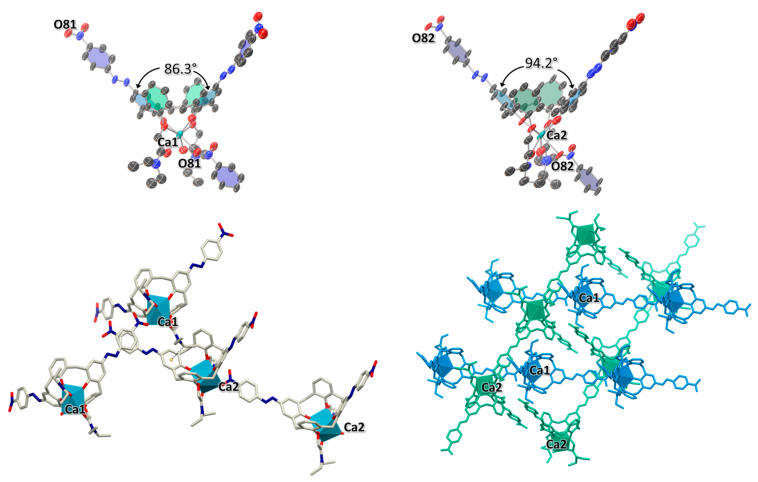
Top: molecular structure of the two complexes contained in the asymmetric unit for **2∙Ca-B** (thermal ellipsoids are depicted at the 30% probability level). Bottom: packing view.

In the asymmetric unit of **2∙Ca-B**, two independent but very similar Ca-calixarene moieties are present, both comprising the Ca^2+^ cation bound to the lower rim of the deprotonated calixarene and without water molecules bound to the metal (Figure 8). The calixarene conformation is analogous to that found in **2·CaCl_2_** and in **2∙Ca-A**. The metal ion adopts a pentagonal bipyramidal geometry and is bound by six oxygen atoms of one calixarene, with the seventh position occupied by a bridging nitro group (Figure S11).

According to the bridging behavior of one of the nitrophenyl groups, two interpenetrated supramolecular chains represent the main packing feature. The bond lengths are consistent with the previously described resonance structure found for **2∙Ca-A**.

## 3. Materials and Methods

### 3.1. General

Solvents and reagents were obtained from commercial sources and used without further purification. Analytical TLC was performed using prepared plates of silica gel (Merck 60 Merck KGaA, Darmstadt, Germany F-254 on aluminum). ^1^H and ^13^C NMR spectra were recorded on a Bruker Billerica, MA, USA AV400 spectrometer. All chemical shifts are reported in parts per million (ppm) using the residual peak of the deuterated solvent, whose values are referred to tetramethylsilane (TMS, δ_TMS_ = 0), as internal standard. ^13^C NMR spectra were performed with proton decoupling. Mass spectra were recorded in ESI mode on a single quadrupole instrument, SQ Detector, Waters (capillary voltage 3.7 kV, cone voltage 30–160 eV, extractor voltage 3 eV, source block temperature 80 °C, desolvation temperature 150 °C, and cone and desolvation gas (N_2_) flow rates 1.6 and 8 L/min, respectively). UV-vis spectra were recorded on a Thermo Scientific Waltham, MA, USA Evolution 260 Bio spectrophotometer. Melting points were determined with a Gallenkamp apparatus.

Diamidocalix[4]arene **1** was synthesized according to a literature procedure [30].

### 3.2. Synthesis of Compound ***2***

A solution of calixarene **1** (0.3 g, 0.46 mmol) in a mixture of THF (25 mL) and pyridine (12 mL) was added dropwise to a solution of 4-nitroaniline (0.25 g, 1.81 mmol) and NaNO_2_ (0.25 g, 3.62 mmol) in 2M HCl (20 mL) cooled to 0 °C with an ice bath. The mixture was stirred at 0 °C for 2 h, then 1M HCl (20 mL) was added, and the resulting precipitate was filtered on a Buchner funnel and washed with methanol. Recrystallization of the solid from CH_2_Cl_2_-methanol yielded compound **2** as a dark orange powder in 36% yield (0.157 g, 0.16 mmol). M.p. > 300 °C (dec.) ^1^H NMR (400 MHz, CDCl_3_): δ 8.34 (d, J = 8.9 Hz, 4H, ArH_o-NO2_), 7.93 (d, J = 8.9 Hz, 4H, ArH _m-NO2_), 7.75 (s, 4H, ArH), 7.12 (d, J = 7.6 Hz, 4H, ArH), 6.87 (t, J = 7.6 Hz, 2H, ArH), 4.92 (s, 4H, OCH_2_), 4.67 (d, J = 13.1 Hz, 4H, ArCH_2_Ar), 3.57–3.50 (m, 8H, ArCH_2_Ar and NCH_2_), 3.42 (q, J = 7.2 Hz, 4H, NCH_2_), 1.31 (t, J = 7.1 Hz, 6H, CH_3_), 1.24 (t, J = 7.1 Hz, 6H, CH_3_). ^13^C NMR (100 MHz, CDCl_3_): δ 167.4 (C=O), 158.2 (ArH), 156.5 (ArH), 153.9 (ArH), 147.8 (ArH), 145.6 (ArH), 133.4 (ArH), 129.5 (ArH), 128.9 (ArH), 125.7 (ArH), 124.8 (ArH), 124.7 (ArH), 122.8 (ArH), 73.1 (OCH_2_), 41.0 (NCH_2_), 40.3 (NCH_2_), 31.7 (ArCH_2_Ar), 14.3 (CH_3_), 13.0 (CH_3_). ESI-MS (m/z): calcd. for C_52_H_52_N_8_O_10_Na^+^ ([M + Na]^+^) 971.37, found 971.62; calcd. for C_52_H_52_N_8_O_10_K^+^ ([M + K]^+^) 987.34, found 987.61. ([M + K^+^]).

### 3.3. X-ray Data Collection

Single crystal data were collected with a Bruker D8 PhotonII area detector diffractometer using microfocus radiation sources (Mo Kα: λ = 0.71073 Å, and Cu Kα: λ = 1.54178 Å). Complete datasets were obtained by merging several series of exposure frames collected at 200 K. An absorption correction was applied with the program SADABS [32] for **2**, **2∙CaCl_2_** and **2∙Ca-A**. Data measured for **2∙Ca-B** were indexed, integrated with a twinning matrix, and scaled using CrysAlis Pro software (version 42.49) accessed on: 12 May 2023 [33]. The structures were solved with ShelxT [34] and refined on F^2^ with full-matrix least squares (ShelxL [35]), using the Olex2 software package (version 1.5) [36]. Non-hydrogen atoms were refined with anisotropic thermal parameters for all compounds.

In **2,** both amide and phenylazo moieties were found disordered over two distinct sites (65:35 occupancy) and refined with a series of SIMU and DFIX restraints. The hydrogen bonded to phenol oxygen was located from the residual electron density map and refined with DFIX restraint.

The asymmetric unit of **2∙CaCl_2_** comprised the calcium cation complexed by the calixarene, one chloride anion positioned on an inversion center, a second chloride anion disordered over two sites having site occupancy factors of 0.5 each, and a third chloride anion disordered over two sites and exchanging hydrogen bond interactions with disordered methanol molecules. According to the refinement, two chloride anions were present overall. One coordinated methanol molecule was split over two sites and refined with DFIX, DANG, and SIMU restraints. The hydrogen atoms bound to the nitrogen atoms of the azo moieties (N5 and N2) could be located from the difference Fourier map, and they were then placed at the calculated positions and refined. The other two partially occupied methanol molecules were pinpointed in the asymmetric unit, while the residual electron density (112 electrons in a volume of 504 Å^3^ for the unit cell) was modelled according to the MASK program. Hence, the masked electron density was taken into account as eight methanol molecules of crystallization for the unit cell.

In **2∙Ca-A**, one of the phenylazo-4-phenol moiety was found disordered over two distinct sites, refined with a 60:40 site occupancy factor and employing a series of SIMU restraints.

In **2∙Ca-B**, owing to poor data quality and twinning, HKL5 was used for the final refinement, and several DFIX, DANG, SIMU, and ISOR restraints and AFIX66 constraints were required. The MASK program was used to model the residual electron density, which corresponded to 290 electrons and 1137 Å^3^ per unit cell. The volume and residual electron count were consistent with the presence of four methanol molecules of crystallization per formula unit.

Crystal data and structure refinement details are provided in the supporting information, Table S1. CCDC 2261451–2261454 contains the supplementary crystallographic data for this paper. These data can be obtained free of charge from the Cambridge Crystallographic Data Centre via www.ccdc.cam.ac.uk/structures accessed on: 12 May 2023.

## 4. Conclusions

A new member of the azocalix[4]arenes family (**2**) was conveniently synthesized in only two steps from commercially available calix[4]arene and studied as a chromoionophore for the Ca^2+^ cation. Thanks to a combination of solution phase and solid-state investigation techniques, we have been able to thoroughly characterize the **2**∙Ca^2+^ complex in light of the azo-phenol–quinone-hydrazone tautomeric equilibration both in neutral and basic conditions. In acetonitrile, the complexation of Ca^2+^ induces a shift of the tautomeric equilibration of the azo-phenol groups towards the quinone-hydrazone form without deprotonation of the ligand. The same structure is observed in the solid state, where, in addition, the counteranion Cl^−^ is hydrogen bonded to the hydrazone NH group. The addition of a weak base such as TEA to the acetonitrile solution of the complex results in the deprotonation of the two quinone-hydrazone moieties, which revert to the azo-phenolate tautomers. The X-ray structure of the neutral complex obtained by treating ligand **2** with Ca(OH)_2_ confirms the restoration of the double N=N bond and the consequent localization of the negative charge on the phenolate ring, in close contact with the cation.

Due to the widespread use of the phenylazophenol moiety as a reporter unit in chromoionophores, we believe our results will be useful for the design of new optical sensors for metal cations.

## Data Availability

Data sets are available from the corresponding author on request.

## Supplementary Materials

The following supporting information can be downloaded at: www.mdpi.com/xxx/s1p; Scheme S1: Resonance forms of azo-phenol moiety; Figure S1: ^1^H NMR spectra of ligand **2** upon addition of Ca(ClO_4_)_2_ and TEA; Table S1: Crystal data and structure refinement details for the measured compounds; Figure S2: Thermal ellipsoid representation of **2**; Figure S3: Thermal ellipsoid representation of **2∙CaCl_2_**; Figure S4: Thermal ellipsoid representation of **2∙Ca-A**; Figure S5: Thermal ellipsoid representation of **2∙Ca-B**; Figure S6: Packing view of **2**; Figure S7: View of intermolecular interactions for **2**; Table S2: Selected intra and intermolecular interactions for **2**; Figure S8: View of **2∙CaCl_2_** showing hydrogen bond interactions involving chloride anions; Figure S9: View of **2∙CaCl_2_** showing supramolecular interactions; Table S3: Selected supramolecular interactions for **2∙CaCl_2_**; Figure S10: View of **2∙Ca-A** showing supramolecular interactions; Table S4: Selected supramolecular interactions for **2∙Ca-A**; Figure S11: Coordination bond lengths and geometries for Ca complexes **2∙CaCl_2_**, **2∙Ca-A** and **2∙Ca-B**; Figure S12: Selected bond lengths for **2** and ***2∙*CaCl_2_**; Figure S13: Selected bond lengths for **2∙Ca-A**.

## Sample Availability

Samples of compound **2** are available from the corresponding author.
