# Supplementary material for: A Combined Solution and Solid-State Study on the Tautomerism of an Azocalix[4]arene Chromoionophore
Source: Molecules. 2023 Jun 12;28(12):4704. doi: 10.3390/molecules28124704 (PMC10304982; doi:10.3390/molecules28124704)
Supplement: Supplementary file 1 [file molecules-28-04704-s001.zip › molecules-2421873-supplementary.pdf]

Supplementary Materials

A Combined Solution and Solid State Study on the Tautomerism of an Azocalix[4]arene Chromoionophore

Laura Baldini *, Davide Balestri, Luciano Marchiò and Alessandro Casnati

Table of Contents

1. Additional Scheme S2
2. NMR Spectroscopy S2
3. X-ray data collection S3
3.1 Integration and refinement details S3
3.2 Asymmetric Units S4
3.3 Packing and weak interactions views S6
3.4 Ca coordination geometries S9
3.5 Selected bond lenghts S10

1. Additional Scheme

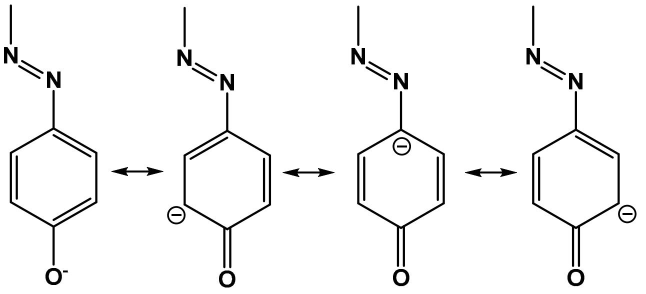

Scheme S1. Resonance forms of azo-phenol moiety.

2. NMR Spectroscopy

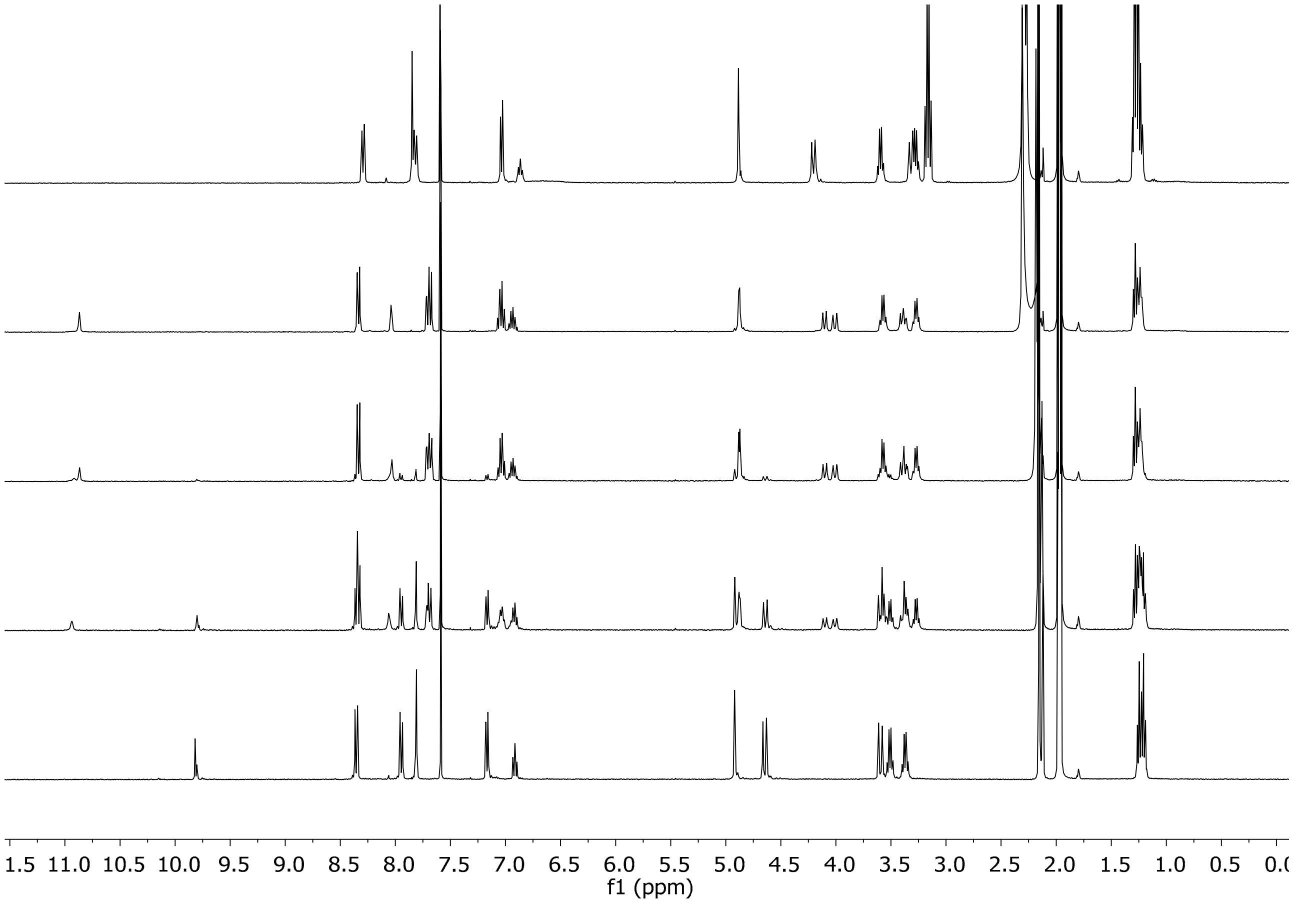

Figure S1. ^{1}H NMR spectra (400 MHz, CD_3CN-$CDCl_3$ 6:1, v/v) of ligand **2** (1.4 mM) upon addition of (from bottom to top) 0, 0.5, 1, 2 equiv. of $Ca(ClO_4)_2$ and 2 equiv. of $Ca(ClO_4)_2$ + 2 equiv. of TEA.

3. X-ray data collection

3.1 Integration and refinement details

Table S1. Crystal data and structure refinement details for the measured compounds

Identification code	**2**	**2·Ca-A**	**2·Ca-B**	**2·CaCl$_2$**
Empirical formula	$C_{52}H_{52}N_8O_{10}$	$C_{104}H_{102}Ca_2N_{16}O_{21}$	$C_{56}H_{66}CaN_8O_{14}$	$C_{57.75}H_{75}CaCl_2N_8O_{15.75}$
Formula weight	949.01	1992.17	1115.24	1244.23
Temperature/K	200.00	200.00	200.00(10)	200.00
Crystal system	monoclinic	monoclinic	triclinic	triclinic
Space group	C2/c	P2$_1$/c	P-1	P-1
a/Å	17.6063(3)	19.9356(10)	17.0705(9)	10.0088(7)
b/Å	19.8737(3)	18.0219(8)	17.0955(11)	11.9844(7)
c/Å	15.4182(2)	29.0503(15)	21.143(3)	27.579(2)
α/°	90	90	89.031(7)	94.075(4)
β/°	117.3680(10)	103.013(4)	83.621(7)	93.844(4)
γ/°	90	90	64.545(6)	102.509(4)
Volume/Å^3	4791.03(13)	10169.1(9)	5533.4(9)	3209.8(4)
Z	4	4	4	2
ρ_{calc}g/cm^3	1.316	1.301	1.339	1.287
μ/mm^{-1}	0.762	1.619	1.592	0.251
F(000)	2000.0	4184.0	2360.0	1315.0
Crystal size/mm^3	0.1 × 0.09 × 0.05	0.09 × 0.05 × 0.03	0.15 × 0.09 × 0.04	0.19 × 0.12 × 0.08
Radiation	CuKα (λ = 1.54178)	CuKα (λ = 1.54178)	CuKα (λ = 1.54184)	MoKα (λ = 0.71073)
2Θ range for data collection/°	7.194 to 140.124	4.55 to 133.188	4.208 to 117.868	4.384 to 50.054
Reflections collected	31983	94653	15944	63268
Independent reflections	4553 [R_{int} = 0.0545, R_{sigma} = 0.0303]	17639 [R_{int} = 0.1594, R_{sigma} = 0.0964]	15944 [R_{int} = 0.131, R_{sigma} = 0.1326]	11171 [R_{int} = 0.1055, R_{sigma} = 0.0783]
Data/restraints/ parameters	4553/217/487	17639/280/1455	15944/1859/1140	11171/125/792
Goodness-of-fit on F^2	1.036	1.042	1.040	1.020
Final R indexes [I>=2σ (I)]	R_1 = 0.0492, wR_2 = 0.1336	R_1 = 0.1066, wR_2 = 0.2457	R_1 = 0.1374, wR_2 = 0.3211	R_1 = 0.0891, wR_2 = 0.2255
Final R indexes [all data]	R_1 = 0.0641, wR_2 = 0.1475	R_1 = 0.2125, wR_2 = 0.3170	R_1 = 0.2159, wR_2 = 0.3639	R_1 = 0.1439, wR_2 = 0.2603
Largest diff. peak/ hole / e Å^{-3}	0.21/-0.15	0.53/-0.47	0.57/-0.36	0.98/-0.47

$R_1 = \Sigma||F_o|-|F_c||/\Sigma|F_o|$, $wR_2 = [\Sigma[w(F_o^2-F_c^2)^2]/\Sigma[w(F_o^2)^2]]^{½}$, $w = 1/[\sigma^2(F_o^2) + (aP)^2 + bP]$, where $P = [max(Fo^2,0) + 2Fc^2]/3$

3.2 Asymmetric Units

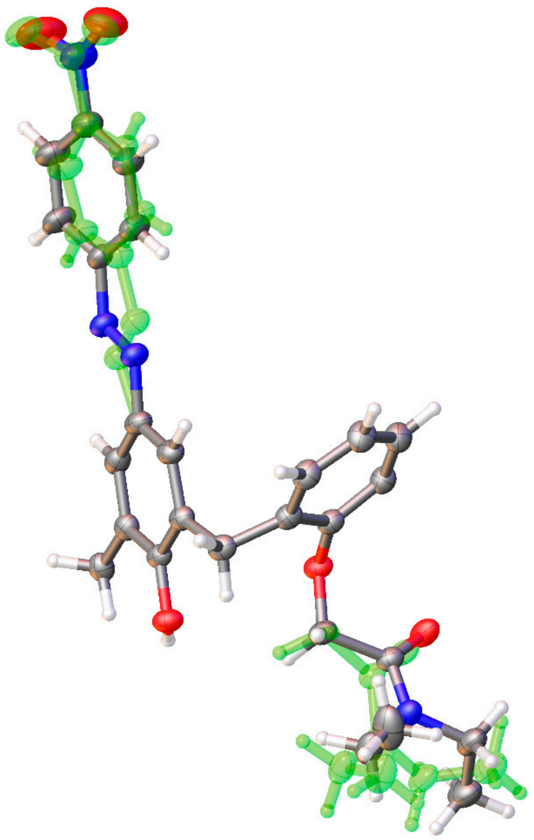

Figure S2. Thermal ellipsoid representation of **2** (30% probability level).The asymmetric unit comprises half calixarene molecule. Disordered moieties are depicted in green-shaded colour.

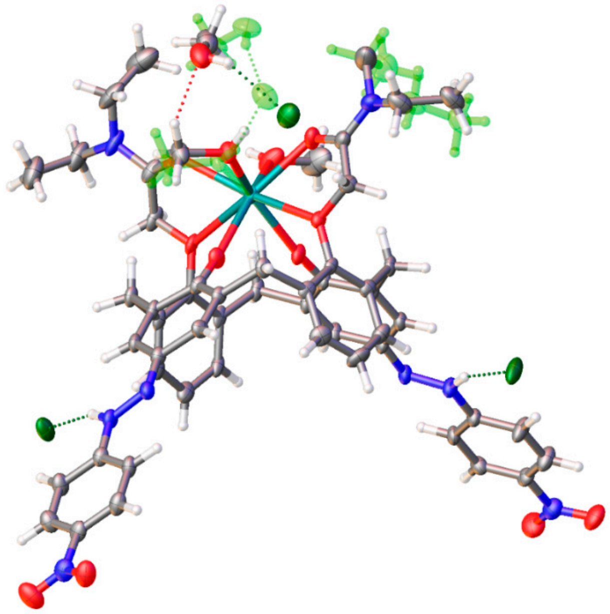

Figure S3. Thermal ellipsoid representation of **2·CaCl₂** (30% probability level). The asymmetric unit comprises one complex molecule, four chloride anions having site occupancy factors of 0.5, two coordinated methanol molecules, and disordered methanol molecules. Disordered moieties are plotted in green-shaded colour.

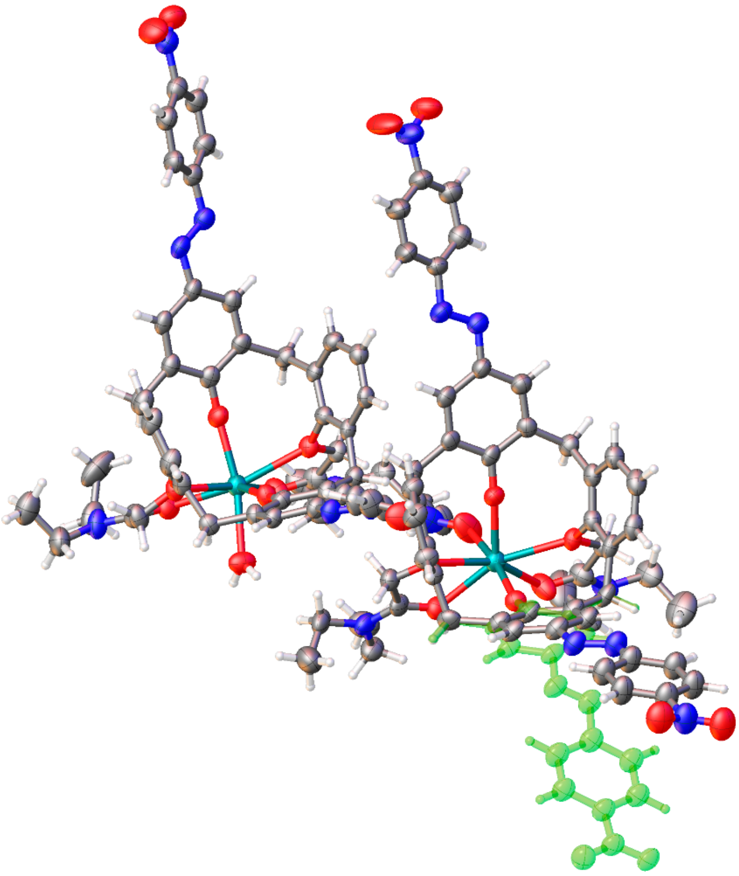

Figure S4. Thermal ellipsoid representation of **2·Ca-A** (30% probability level).The asymmetric unit comprises two complex molecule. Disordered moieties are plotted in green-shaded colour.

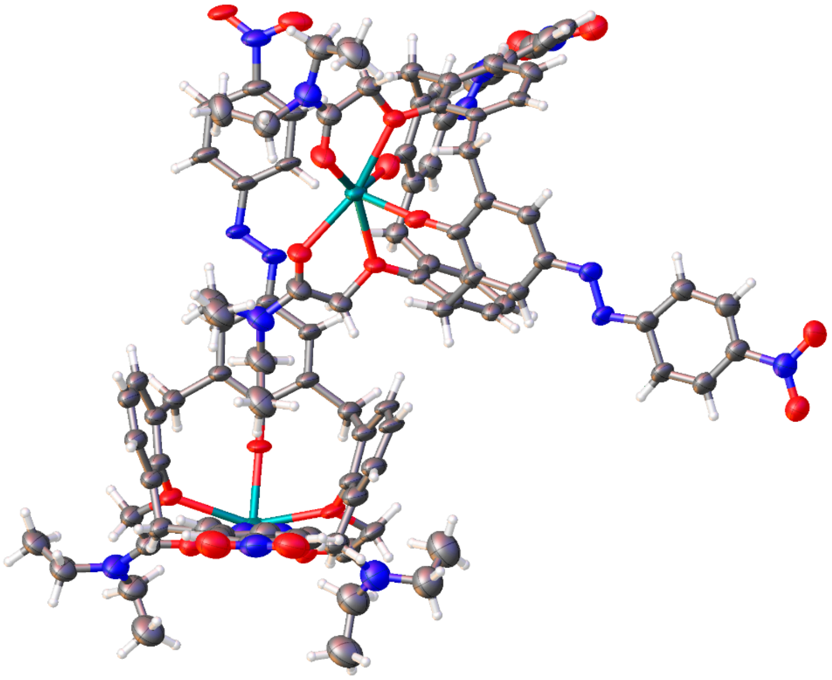

Figure S5. Thermal ellipsoid representation of **2·Ca-B** (30% probability level). The asymmetric unit comprises two complex molecule. Disordered moieties are plotted in green-shaded colour.

3.3 Packing and weak interactions views

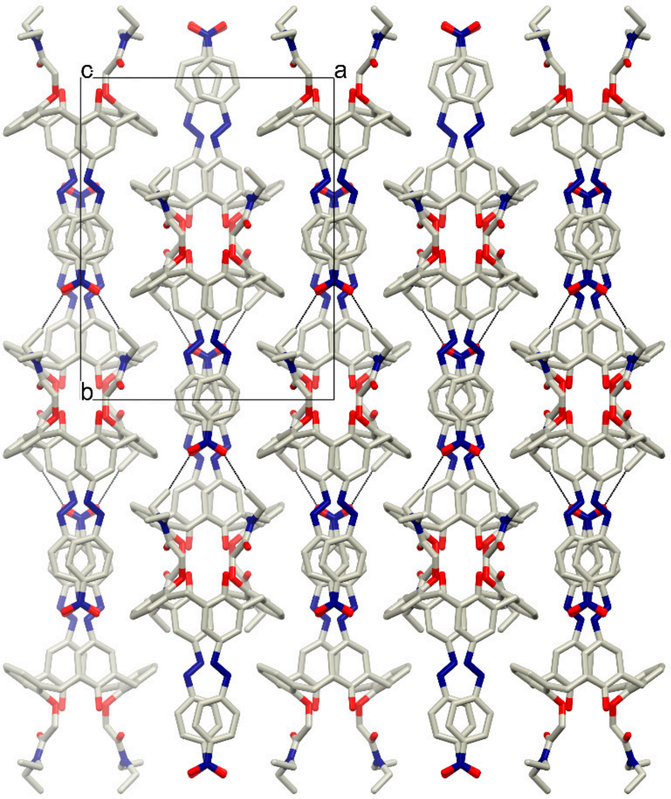

Figure S6. Packing view of **2** along crystallographic axis *c* and weak intermolecular interactions between nitro and methyl group belonging to neighboring calixarene molecules.

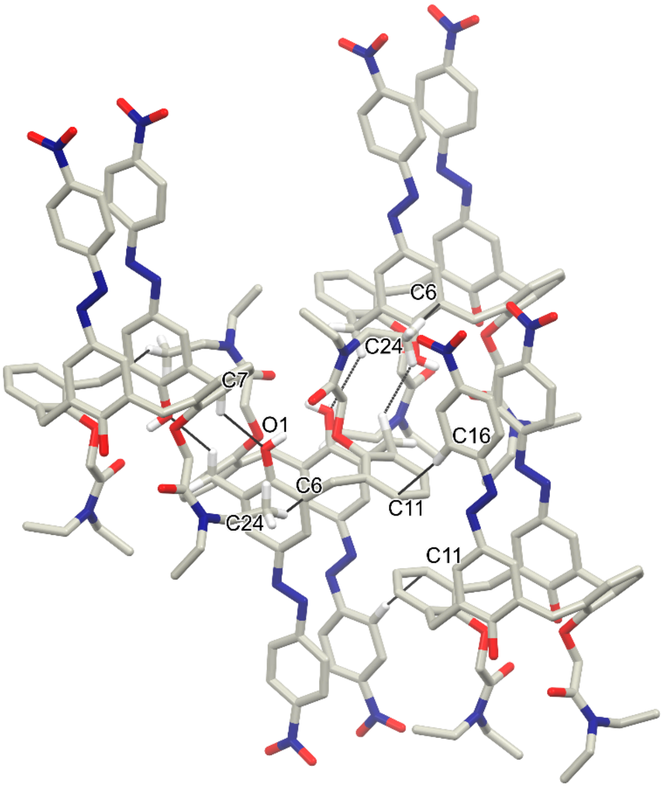

Figure S7. View of CH···π and CH···O intermolecular interactions for **2**.

Table S2. Selected intra and intermolecular interactions (Å) for **2.**

Hydrogen Bonds		***Weak Contacts***	
Interaction	*Distance*	*Interaction*	*Distance*
O1···O5	2.631(4)	O1···C7	3.364(2)
O1···O5A	2.613(8)	C53···O10	3.536(9)
		C26···O3	2.977(6)
		C24···O4	3.43(1)
		C16···C11	3.665(5)
		C24···C6	3.558(6)

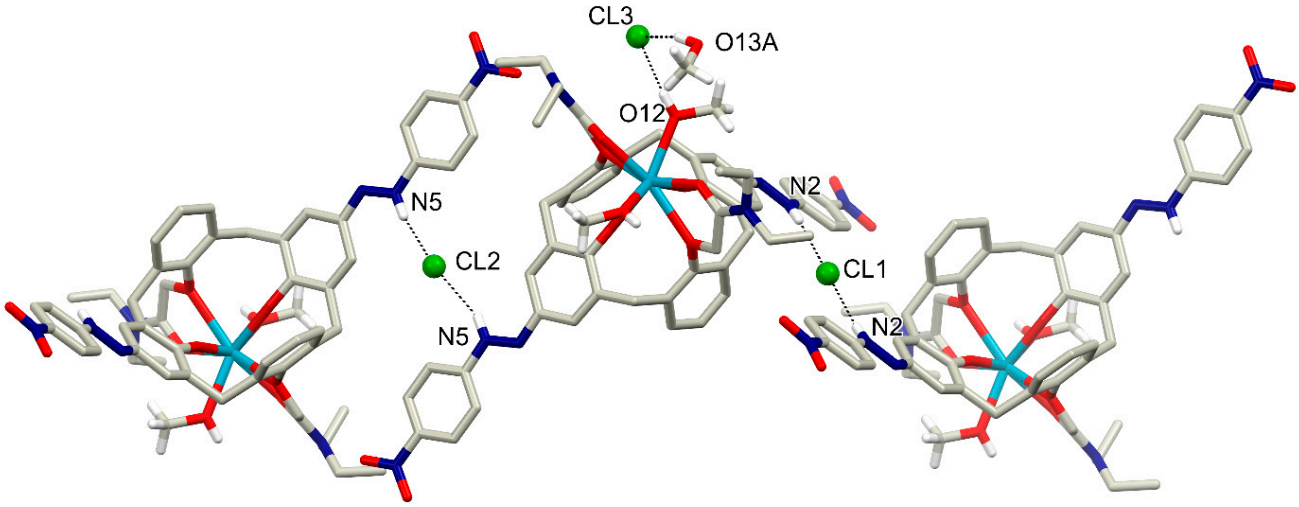

Figure S8. View of **2·CaCl$_2$** showing hydrogen bond interactions involving chloride anions.

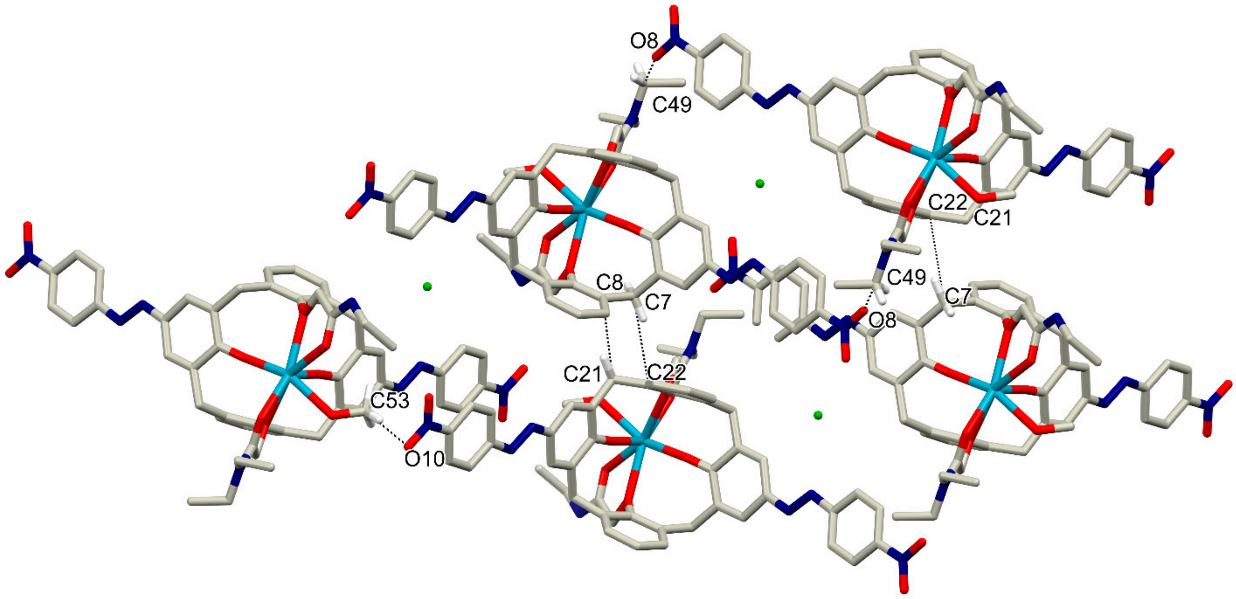

Figure S9. View of **2·CaCl$_2$** showing CH···π and CH···O supramolecular interactions.

Table S3. Selected supramolecular interactions (Å) for **2·CaCl$_2$**

Hydrogen Bonds		*Weak Contacts*	
Interaction	*Distance*	*Interaction*	*Distance*
N2···Cl1	3.241	C49···O8	3.284(9)
N5···Cl2	3.283(7)	C53···O10	3.536(9)
O12A···Cl3A	3.07(3)	C7···Cl1	3.653(8)
O12···Cl3	3.26(3)	C8···C21	3.672(2)
O13···Cl3A	3.18(1)		
O13A···Cl3	3.29(2)		

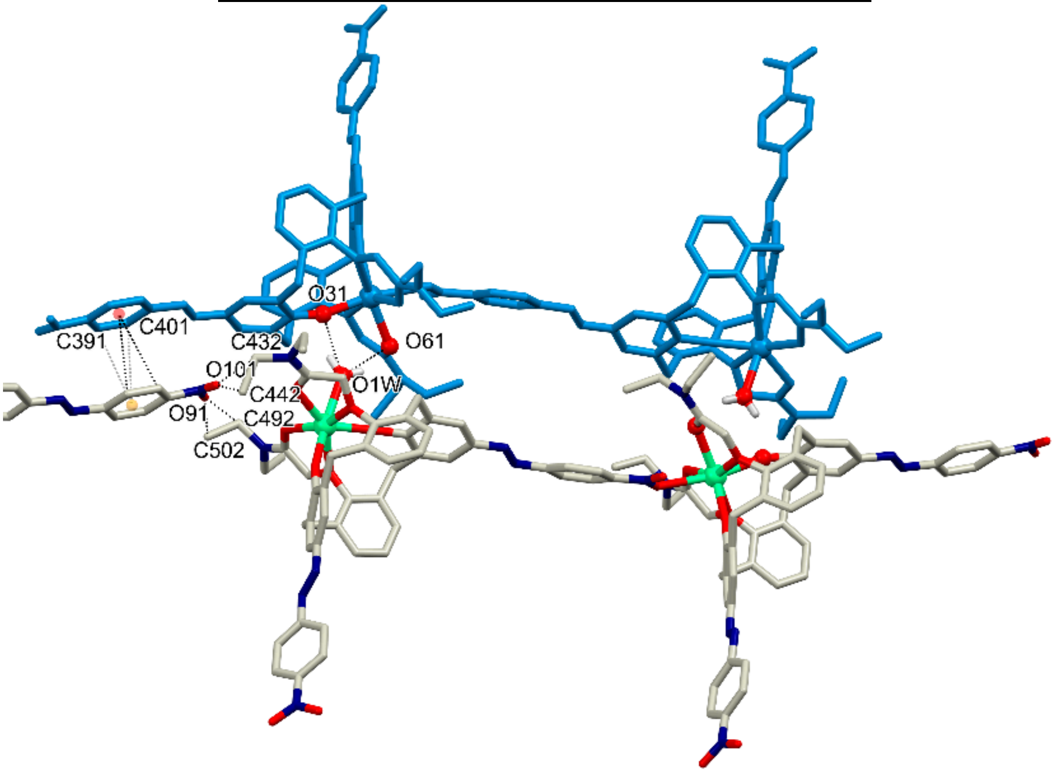

Figure S10. View of **2·Ca-A** showing supramolecular interactions.

Table S4. Selected supramolecular interactions (Å) for **2·Ca-A.**

Hydrogen Bonds		*Weak Contacts*	
Interaction	*Distance*	*Interaction*	*Distance*
O1W···O31	2.745(9)	O101···C432	2.95(2)
O1W·· O61	3.062(8)	C91···C492	3.10(2)
		Centroid···C391	3.586
		Centroid···C401	3.506

3.4 Ca coordination geometries

**2·CaCl$_2$**			
Ca-O2	2.604(3)	Ca-O5	2.391(4)
Ca-O4	2.647(4)	Ca-O11	2.453(4)
Ca-O3	2.346(4)	Ca-O12	2.49(2)
Ca-O1	2.370(4)	Ca-O12A	2.37(3)
Ca-O6	2.355(4)		

**2·Ca-A**			
Ca1-O11	2.214(5)	Ca2-O12	2.251(5)
Ca1-O21	2.488(5)	Ca2-O42	2.518(5)
Ca1-O41	2.491(5)	Ca2-O32	2.212(6)
Ca1-O31	2.167(6)	Ca2-O22	2.450(5)
Ca1-O61	2.427(6)	Ca2-O1w	2.391(6)
Ca1-O51	2.373(6)	Ca2-O62	2.377(5)
Ca1-O72	2.576(7)	Ca2_O52	2.427(6)

**2·Ca-B**			
Ca1-O11	2.480(7)	Ca2-O12	2.535(9)
Ca1-O31	2.495(8)	Ca2-O42	2.246(8)
Ca1-O41	2.121(8)	Ca2-O32	2.363(10)
Ca1-O21	2.186(11)	Ca2-O52	2.342(11)
Ca1-O51	2.326(10)	Ca2-O82	2.557(7)
Ca1-O81	2.503(11)	Ca2-O62	2.212(12)
Ca1-O61	2.343(9)	Ca2-O22	2.187(14)

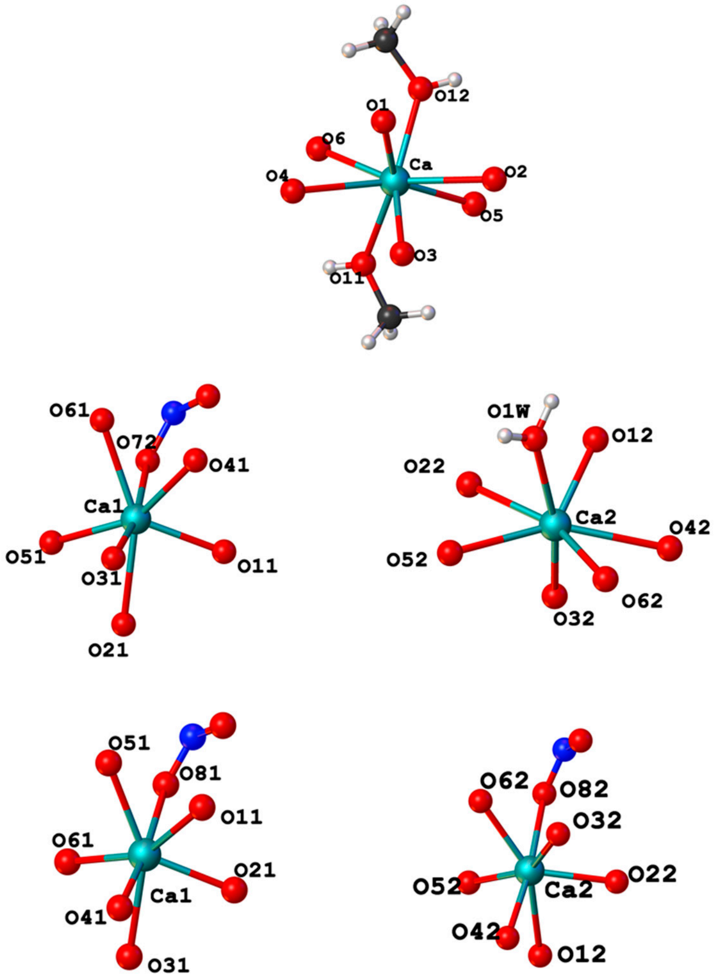

Figure S11. Coordination bond lengths (Å) and geometries for Ca complexes **2·CaCl$_2$**, **2·Ca-A** and **2·Ca-B.**

3.5 Selected bond lenghts

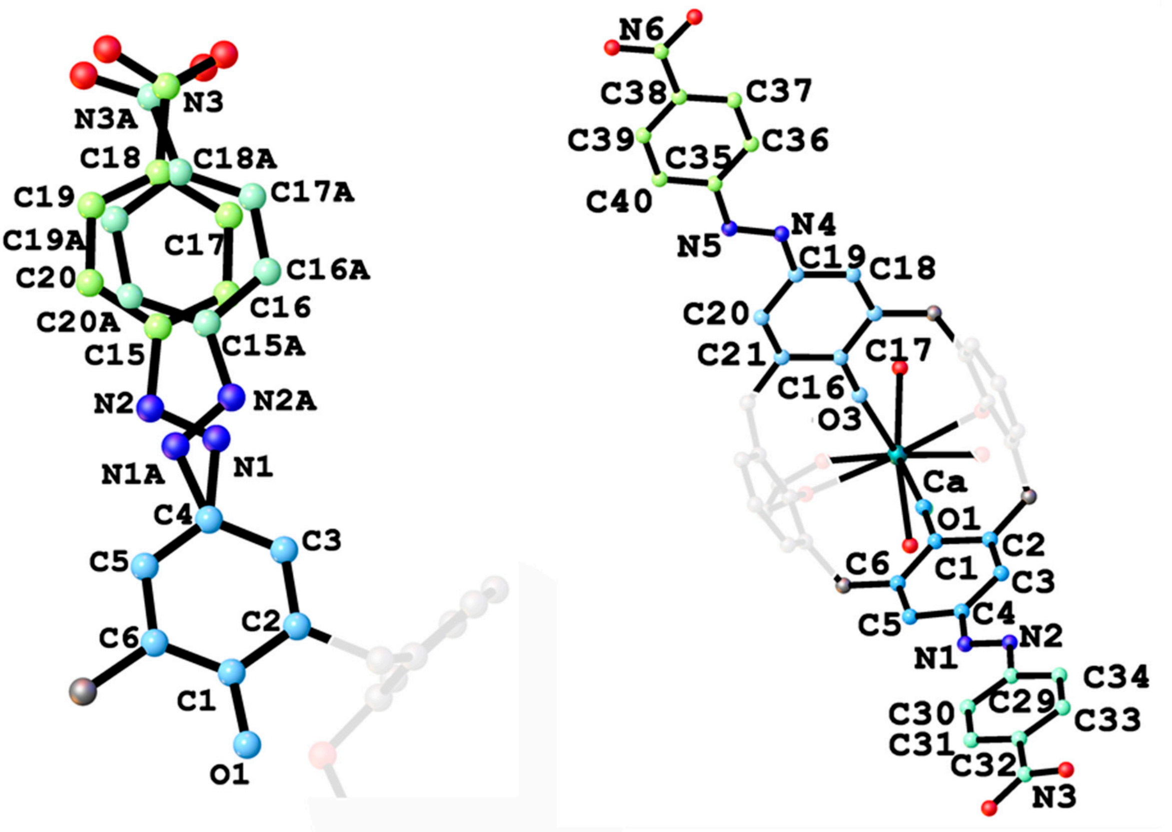

	2				2·$CaCl_2$			
O-phenyl-azo	O1-C1	1.347(2)			O1-C1	1.229(6)	O3-C16	1.240(6)
	C1-C2	1.405(3)			C1-C2	1.492(7)	C16-C17	1.457(7)
	C2-C3	1.380(3)			C2-C3	1.332(7)	C17-C18	1.339(7)
	C3-C4	1.384(3)			C3-C4	1.441(7)	C18-C19	1.445(7)
	C4-C5	1.390(3)			C4-C5	1.460(7)	C19-C20	1.447(7)
	C5-C6	1.383(2)			C5-C6	1.343(7)	C20-C21	1.351(7)
	C6-C1	1.411(2)			C6-C1	1.454(7)	C21-C16	1.476(6)
	C4-N1	1.458(4)			C4-N1	1.321(7)	C19-N4	1.312(6)
	N1-N2	1.240(4)	N1A-N2A	1.291(7)	N1-N2	1.334(6)	N4-N5	1.342(6)
N-phenyl-nitro	N2-C15	1.431(4)	N2A-C15A	1.384(8)	N2-C29	1.410(7)	N5-C35	1.384(6)
	C15-C16	1.376(7)	C15A-C16A	1.413(11)	C29-C30	1.382(7)	C35-C36	1.379(7)
	C16-C17	1.401(7)	C16A-C17A	1.345(12)	C30-C31	1.383(8)	C36-C37	1.380(7)
	C17-C18	1.392(8)	C17A-C18A	1.321(14)	C31-C32	1.386(8)	C37-C38	1.397(8)
	C18-C19	1.346(8)	C18A-C19A	1.406(16)	C32-C33	1.373(8)	C38-C39	1.350(8)
	C19-C20	1.392(8)	C19A-C20A	1.395(13)	C33-C34	1.372(8)	C39-C40	1.360(8)
	C20-C15	1.381(6)	C20A-C15A	1.393(10)	C34-C29	1.397(8)	C40-C35	1.403(8)
	C18-N3	1.507(10)	C18A-N3A	1.488(19)	C32-N3	1.474(8)	C38-N6	1.450(7)

Figure S12. Selected bond lengths (Å) for **2** and **2·$CaCl_2$**

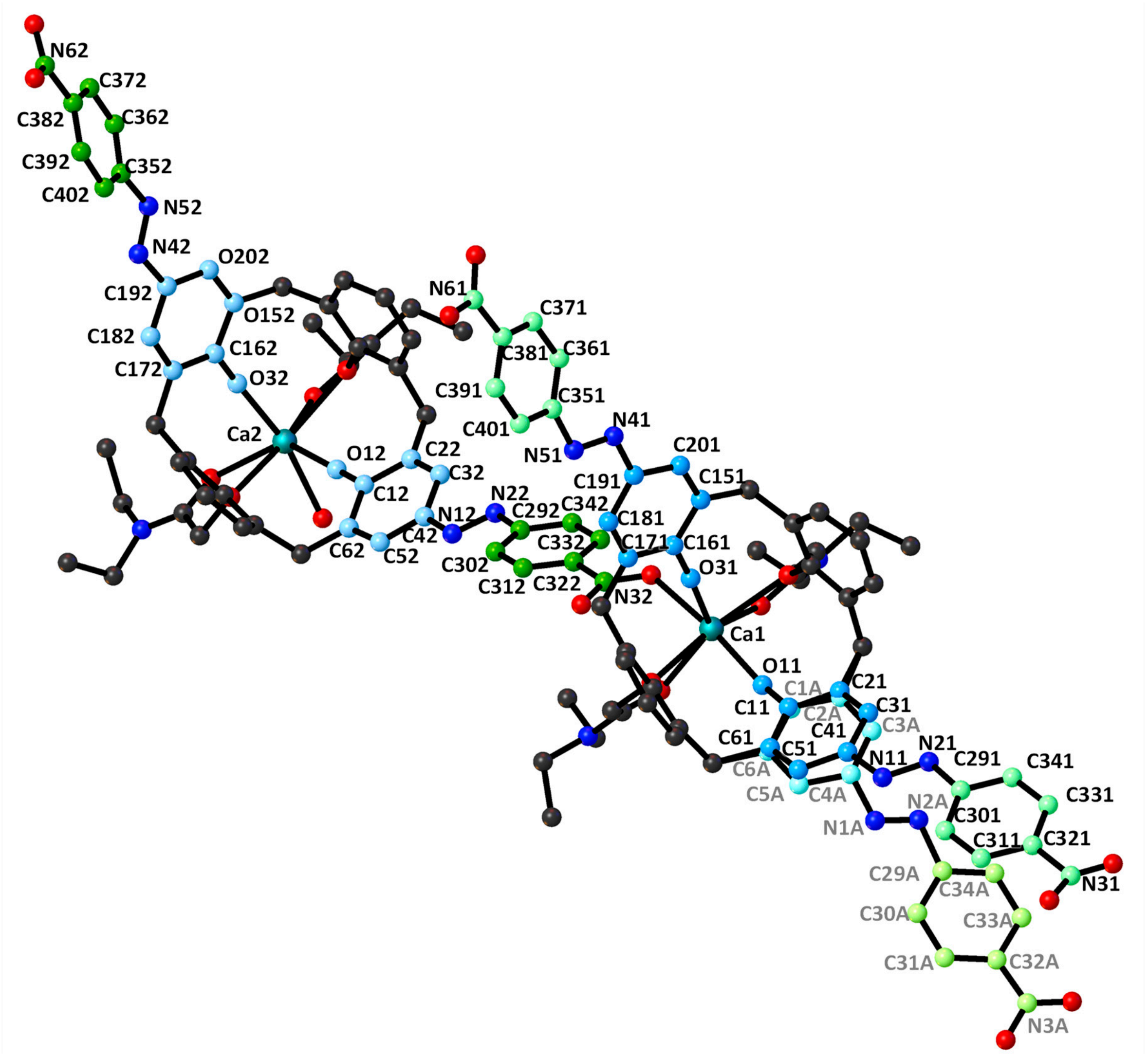

	Ca1 complex						*Ca2 complex*			
**O-phenyl-azo**	O11-C11	1.313(12)	O11-C1A	1.313(10)	O31-C161	1.295(9)	O12-C12	1.287(8)	O32-C162	1.285(9)
	C11-C21	1.403(15)	C1A-C2A	1.403(13)	C151-C161	1.436(10)	C12-C22	1.434(10)	C152-C162	1.417(10)
	C21-C31	1.37(2)	C2A-C3A	1.421(16)	C161-C171	1.418(10)	C22-C32	1.381(9)	C162-C172	1.437(10)
	C31-C41	1.43(3)	C3A-C4A	1.407(19)	C171-C181	1.383(10)	C32-C42	1.384(10)	C172-C182	1.363(10)
	C41-C51	1.41(3)	C4A-C5A	1.365(15)	C181-C191	1.400(10)	C42-C52	1.425(10)	C182-C192	1.401(11)
	C51-C61	1.375(16)	C5A-C6A	1.365(15)	C191-C201	1.408(11)	C52-C62	1.355(9)	C192-C202	1.401(11)
	C61-C11	1.440(14)	C6A-C1A	1.437(13)	C201-C151	1.387(11)	C62-C12	1.427(10)	C202-C152	1.371(11)
	C41-N11	1.39(2)	C4A-N1A	1.416(16)	C191-N41	1.404(10)	C42-N12	1.393(9)	C192-N42	1.401(10)
	N11-N21	1.27(2)	N1A-N2A	1.211(12)	N41-N51	1.283(9)	N12-N22	1.270(8)	N42-N52	1.289(9)
**N-phenyl-nitro**	N21-C291	1.429(17)	N2A-C29A	1.473(14)	N51-C351	1.422(10)	N22-C292	1.415(10)	N52-C352	1.426(10)
	C291-C301	1.37(3)	C29A-C30A	1.44(2)	C351-C361	1.364(12)	C292-C302	1.370(12)	C352-C362	1.372(11)
	C301-C311	1.422(18)	C30A-C31A	1.43(2)	C361-C371	1.395(12)	C302-C312	1.397(12)	C362-C372	1.401(12)
	C311-C321	1.33(3)	C31A-C32A	1.37(2)	C371-C381	1.334(12)	C312-C322	1.338(13)	C372-C382	1.401(12)
	C321-C331	1.37(3)	C32A-C33A	1.37(3)	C381-C391	1.383(13)	C322-C332	1.376(14)	C382-C392	1.379(12)
	C331-C341	1.43(3)	C33A-C34A	1.36(2)	C391-C401	1.366(12)	C332-C342	1.385(12)	C392-C402	1.381(12)
	C341-C291	1.34(3)	C34A-C29A	1.34(2)	C401-C351	1.383(12)	C342-C292	1.365(12)	C402-C352	1.384(11)
	C321-N31	1.479(17)	C32A-N3A	1.467(19)	C381-N61	1.469(12)	C322-N32	1.461(12)	C382-N62	1.493(12)

Figure S13. Selected bond lengths (Å) for **2·Ca-A.**
